# Supplementary material for: BIN2 phosphorylates the Thr280 of CO to restrict its function in promoting Arabidopsis flowering
Source: Front Plant Sci. 2023 Jan 30;14:1068949. doi: 10.3389/fpls.2023.1068949 (PMC9923014; doi:10.3389/fpls.2023.1068949)
Supplement: Supplementary file 1 [file DataSheet_1.pdf]

Figure 1A

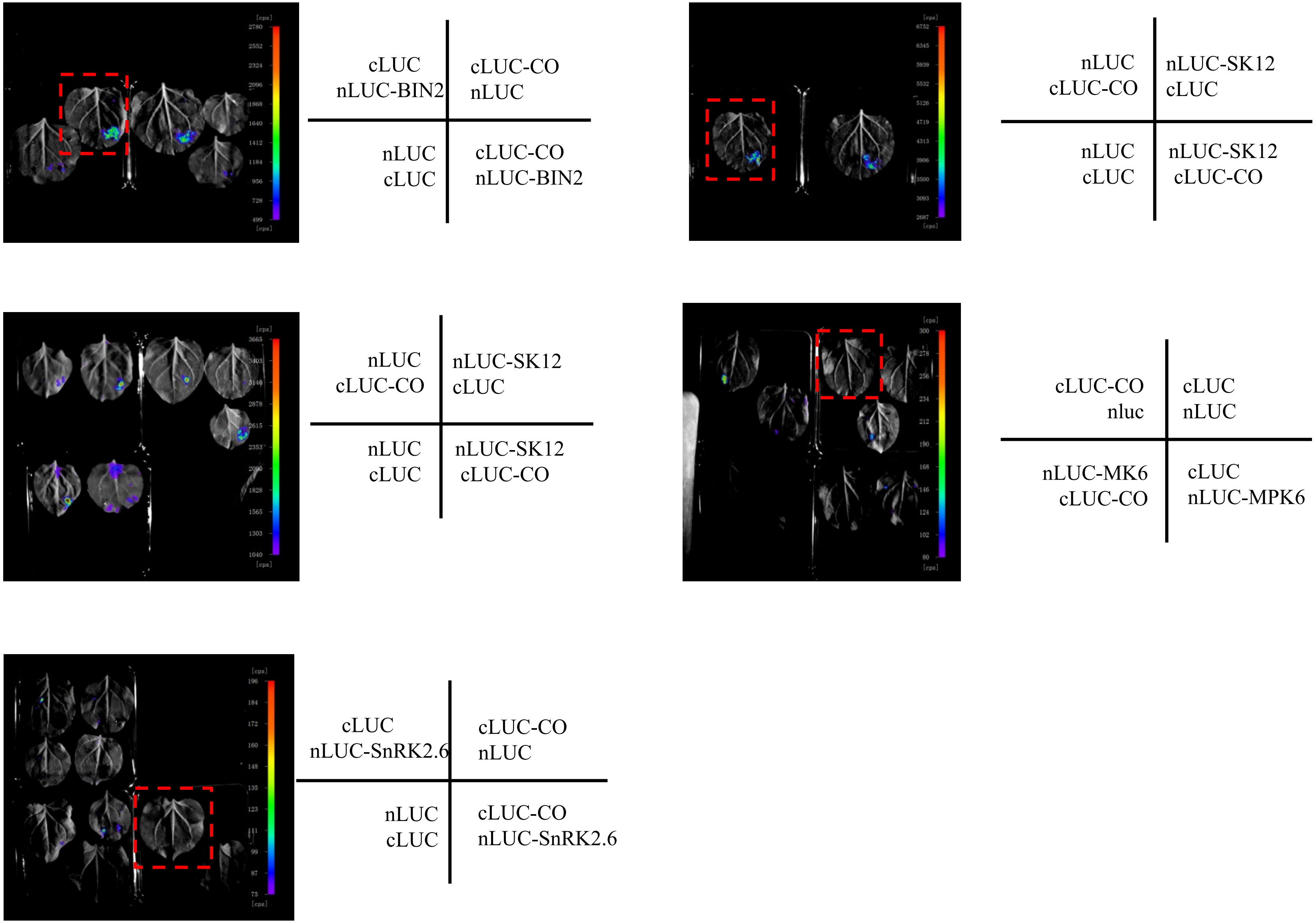
cLUC
nLUC-BIN2
cLUC-CO
nLUC
nLUC
cLUC
cLUC-CO
nLUC-BIN2
nLUC
cLUC-CO
nLUC-SK12
cLUC
nLUC
cLUC
nLUC-SK12
cLUC-CO
nLUC
cLUC-CO
nLUC-SK12
cLUC
nLUC
cLUC
nLUC-SK12
cLUC-CO
cLUC-CO
nluc
cLUC
nLUC
nLUC-MK6
cLUC-CO
cLUC
nLUC-MPK6
cLUC
nLUC-SnRK2.6
cLUC-CO
nLUC
nLUC
cLUC
cLUC-CO
nLUC-SnRK2.6

Figure 1B

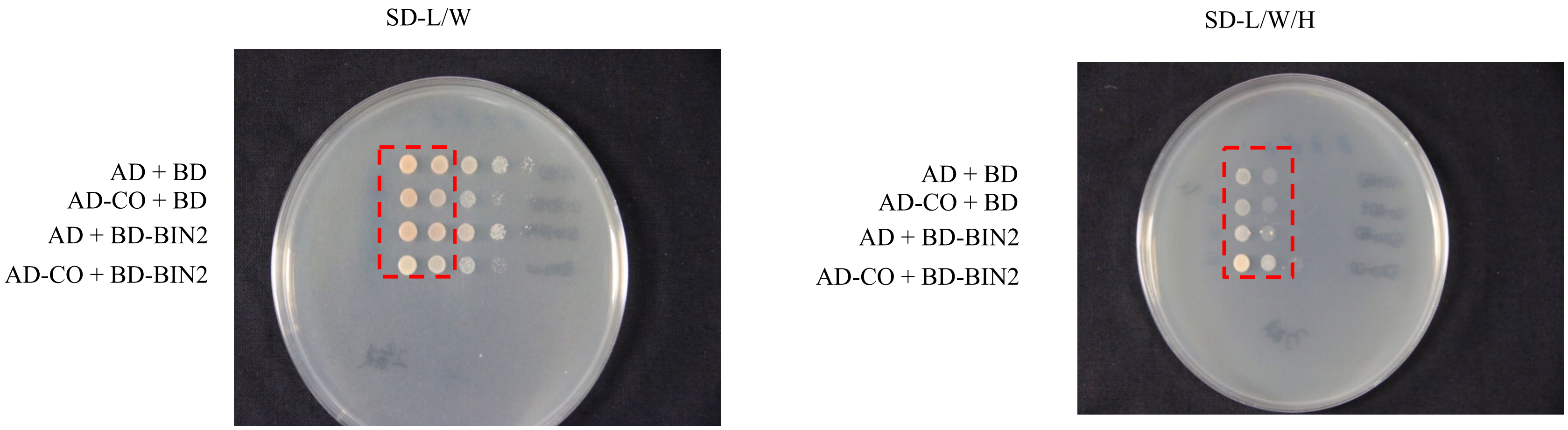

Figure 1C

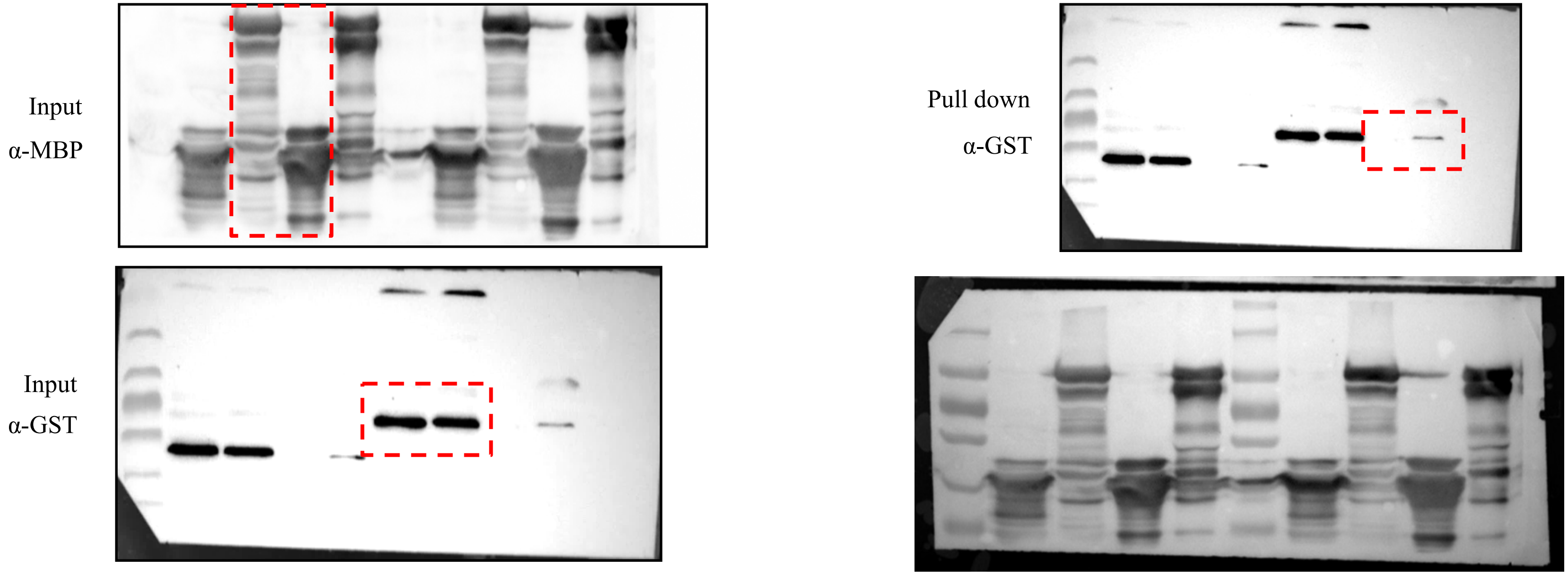

Figure 1D

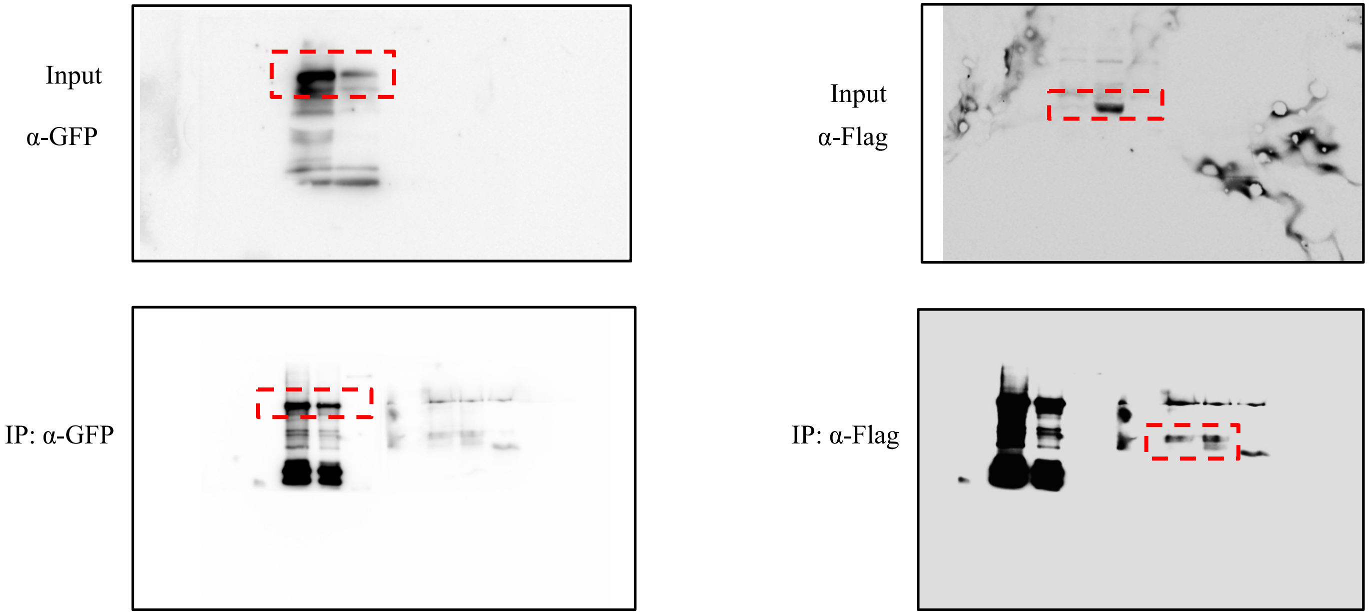

Figure 1F

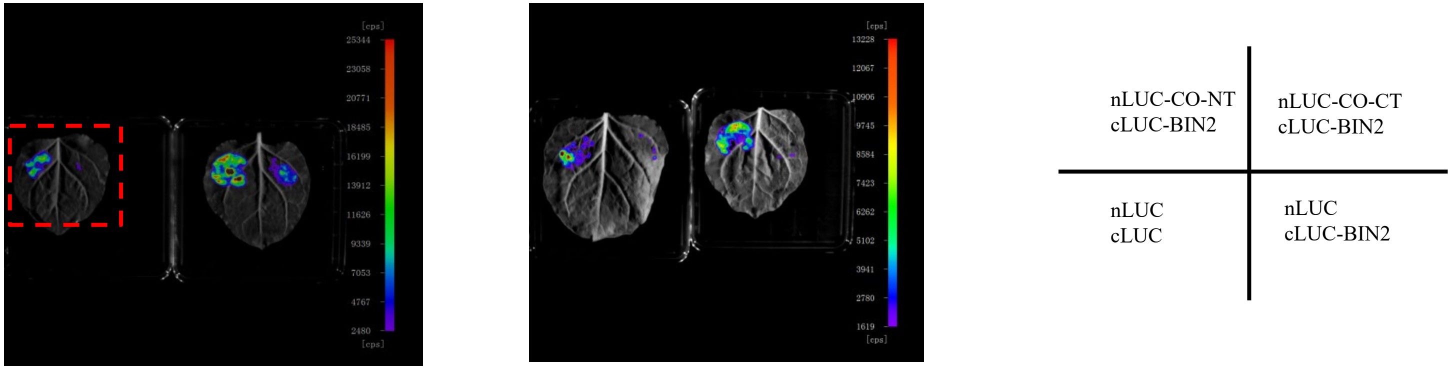

Figure 1G

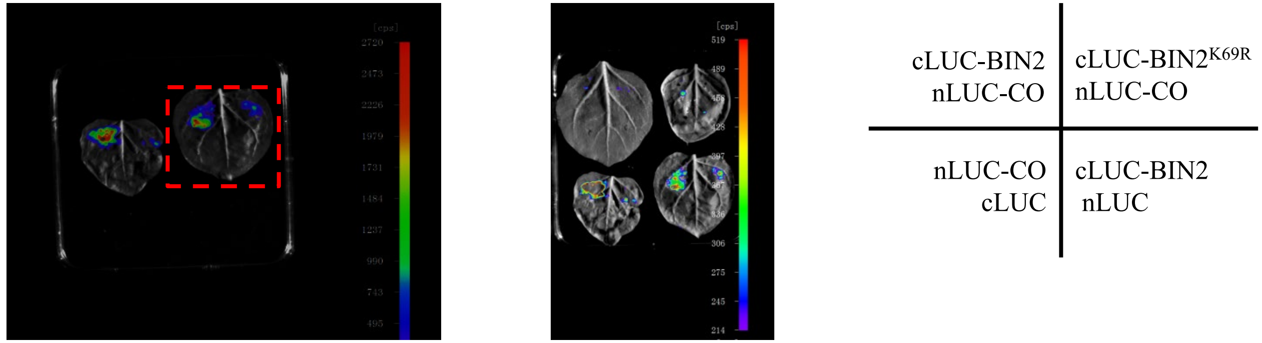

Figure 2A

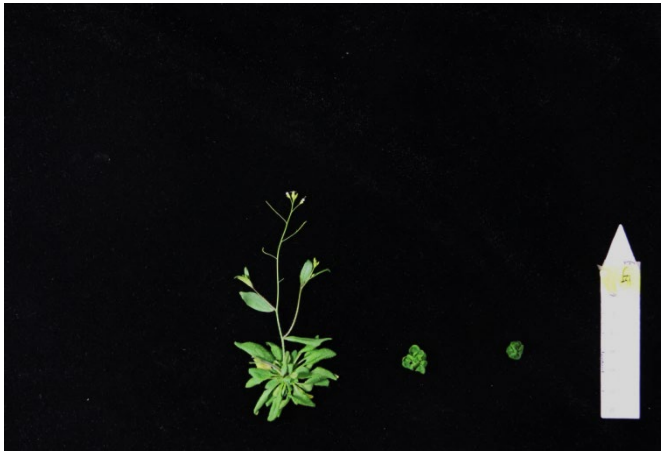

Figure 2D

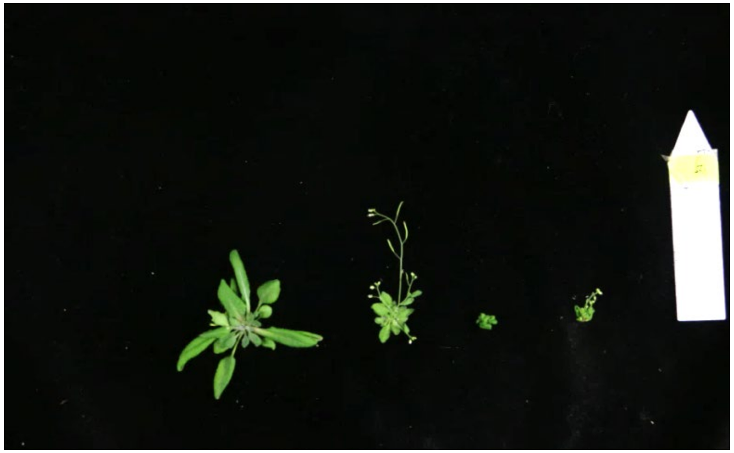

Figure 3A

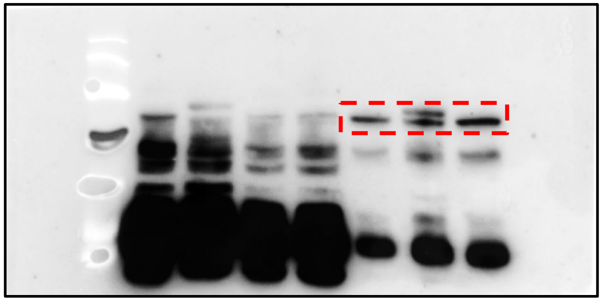

α-GST

Figure 3B

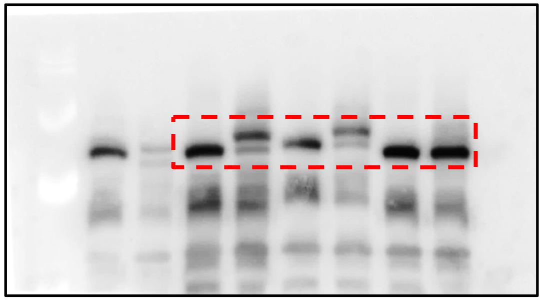

α-GST

Figure 3C

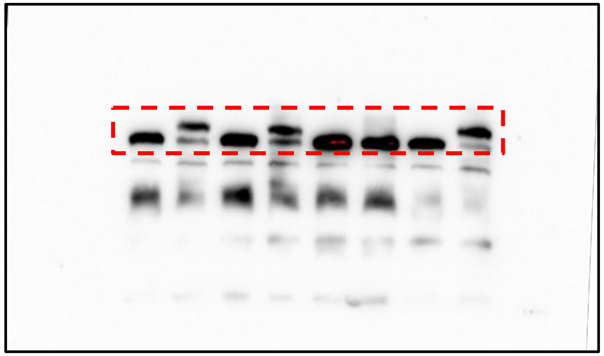

α-GST

Figure 3D

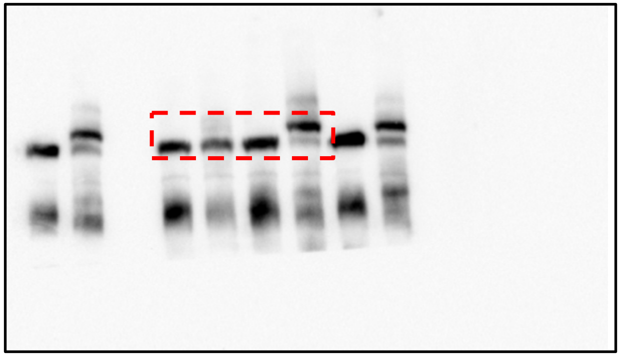

α-GST

Figure 4A

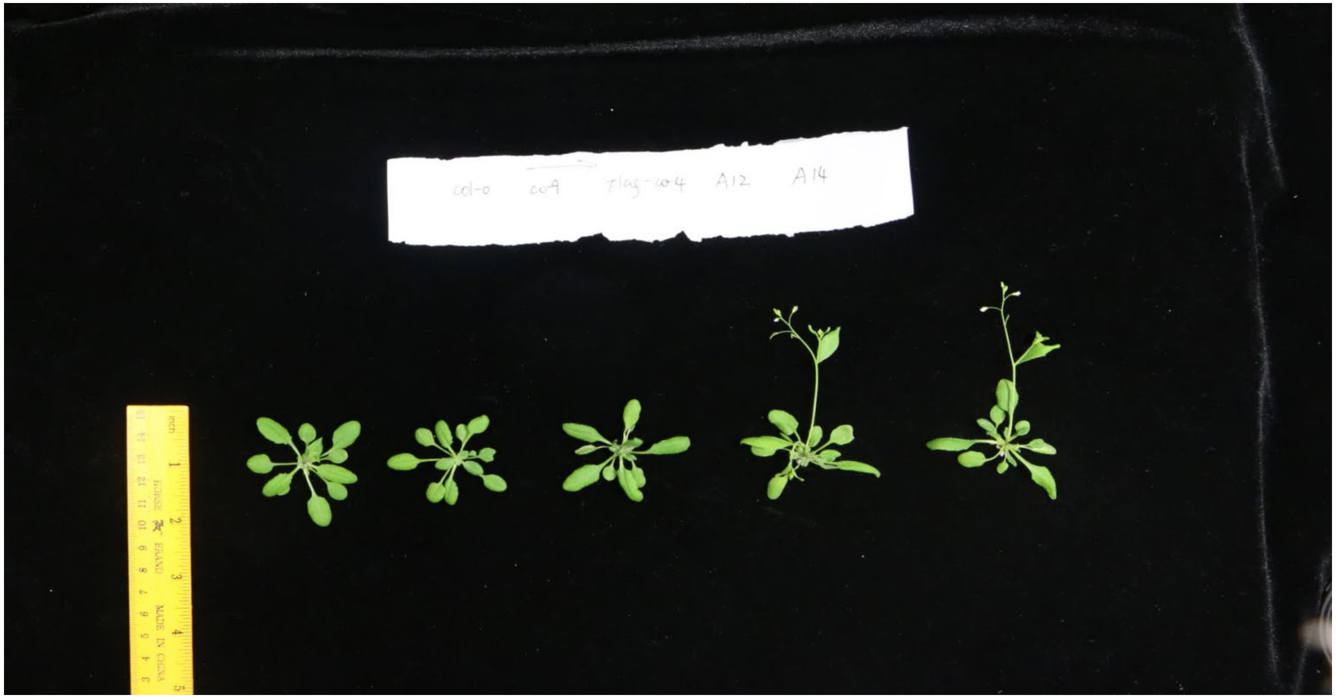

Figure 4F

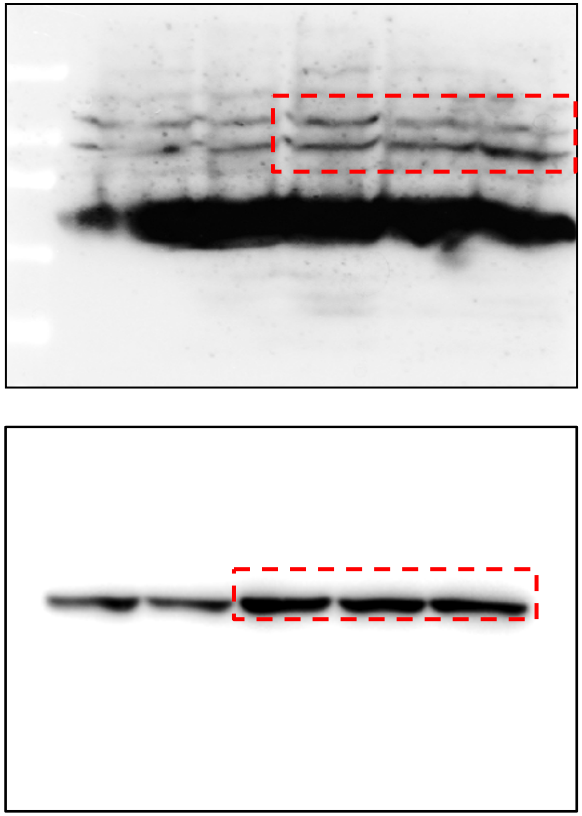

α-Flag

α-Actin

Figure 5A

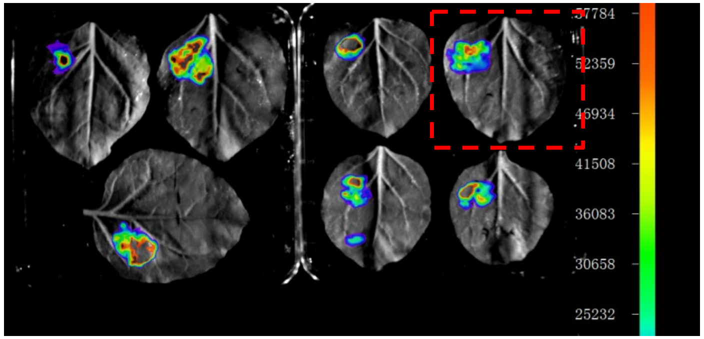

nLUC-CO<br>cLUC-CO	nLUC-CO<br>cLUC
nLUC<br>cLUC	cLUC-CO<br>nLUC

Figure 5B

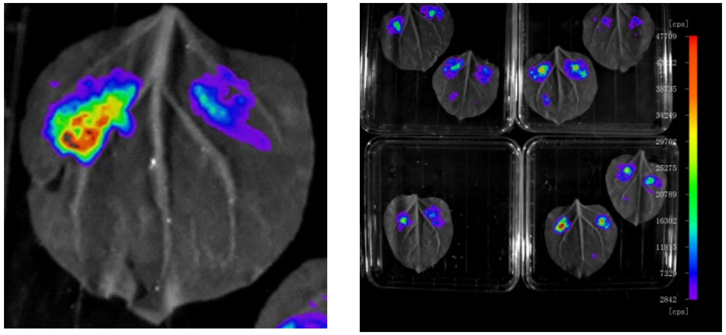

nLUC-CO-NT<br>cLUC-CO-NT<br>YFP	nLUC-CO-NT<br>cLUC-CO-NT<br>BIN2-Flag
nLUC<br>cLUC-CO-NT<br>YFP	cLUC<br>nLUC-CO-NT<br>YFP

Figure 5E

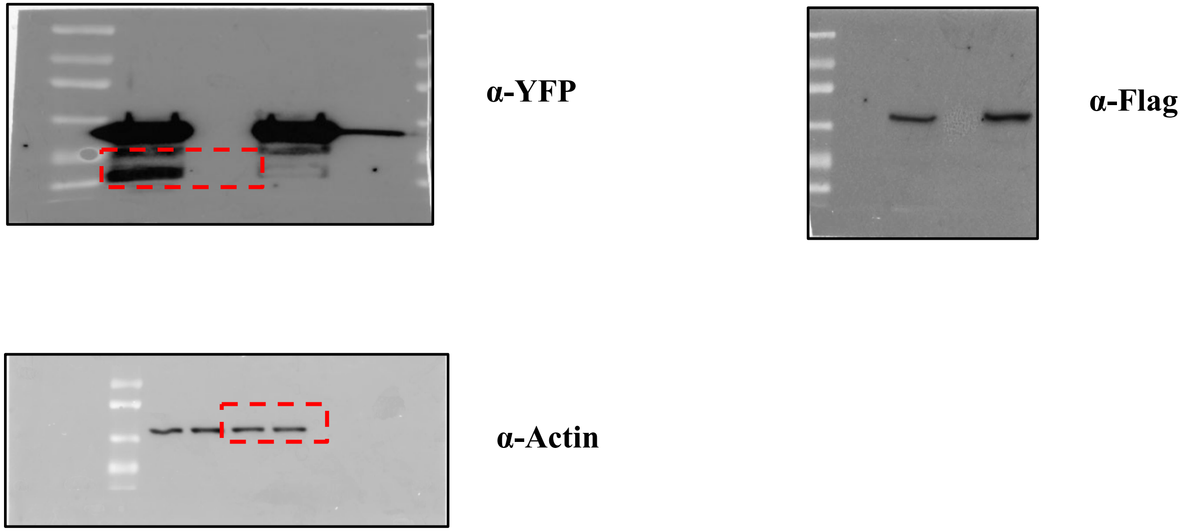

Supplemental Figure 2

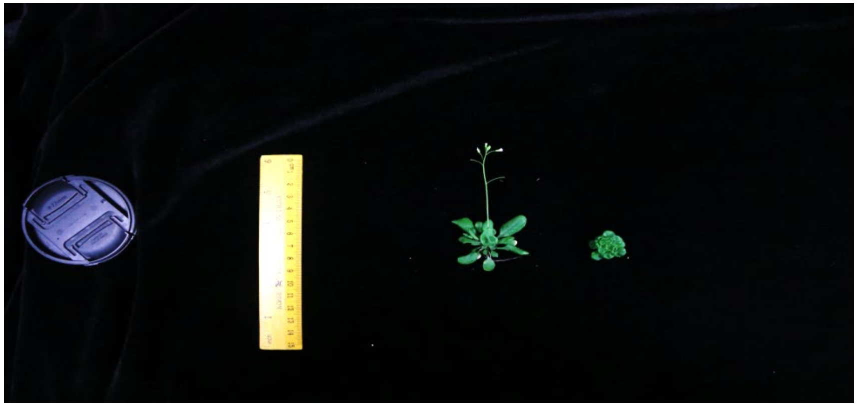

Supplemental Figure 4

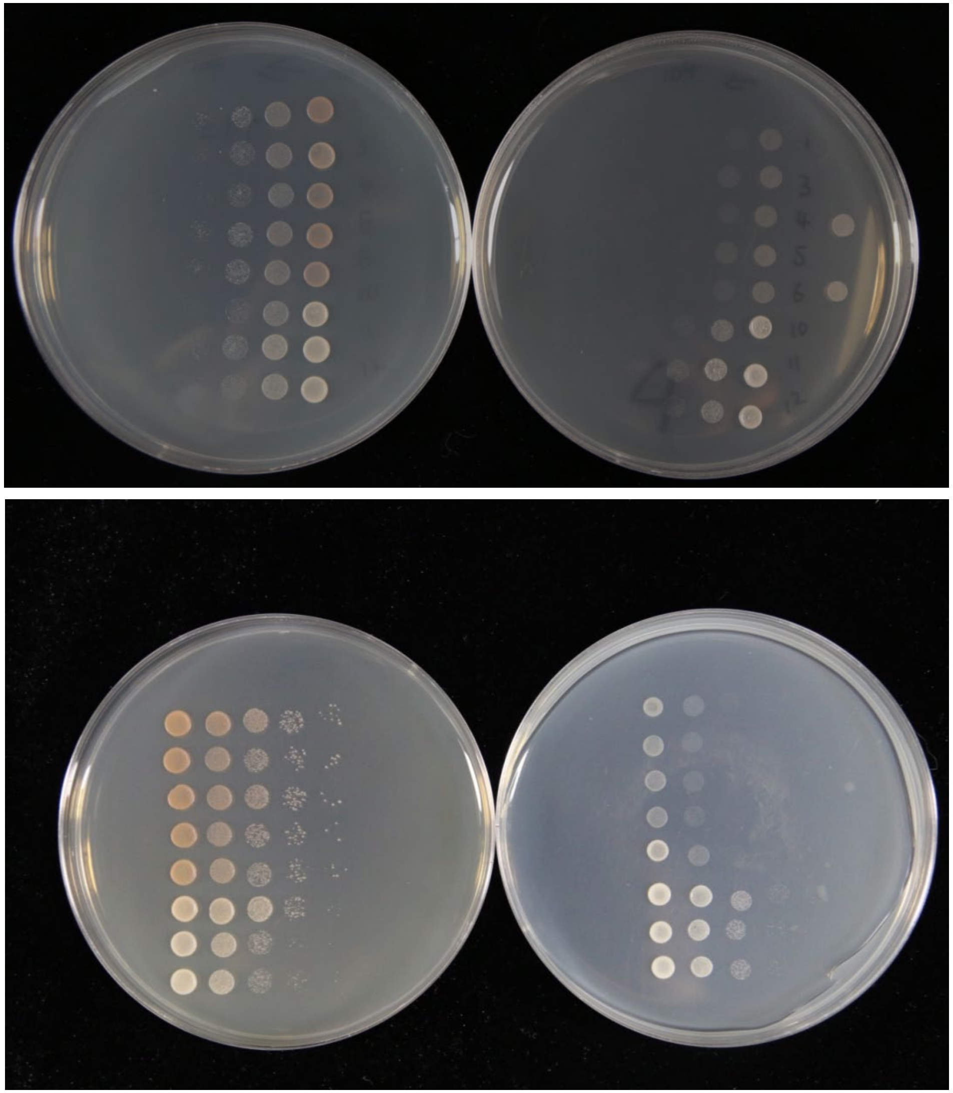

Supplemental Figure 6

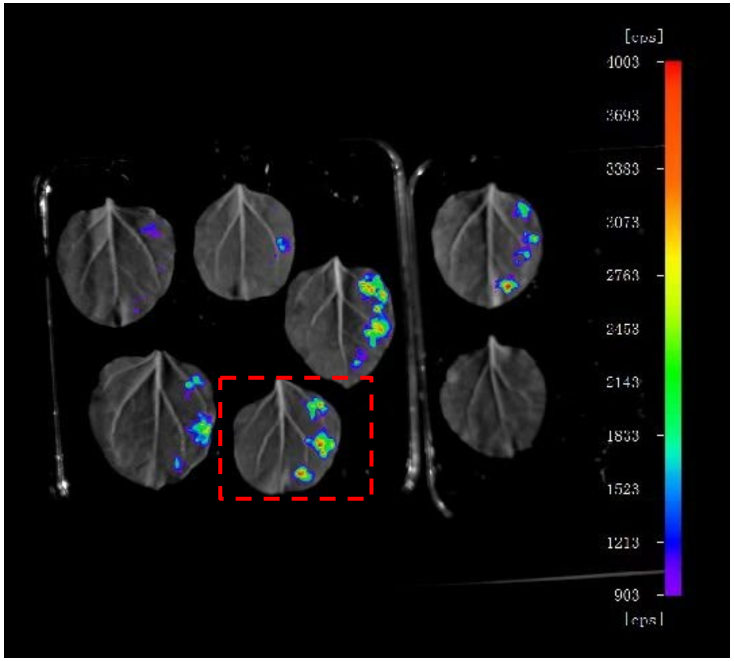

cLUC-CO<br>nLUC	cLUC-CO<br>nLUC-CO
cLUC-CO^{T280A}<br>nLUC	cLUC-CO^{T280A}<br>nLUC-CO^{T280A}
cLUC-CO^{T280D}<br>nLUC	cLUC-CO^{T280D}<br>nLUC-CO^{T280D}
