# Supplementary figures and images for: BIN2 phosphorylates the Thr280 of CO to restrict its function in promoting Arabidopsis flowering
Source: Front Plant Sci. 2023 Jan 30;14:1068949. doi: 10.3389/fpls.2023.1068949 (PMC9923014; doi:10.3389/fpls.2023.1068949)

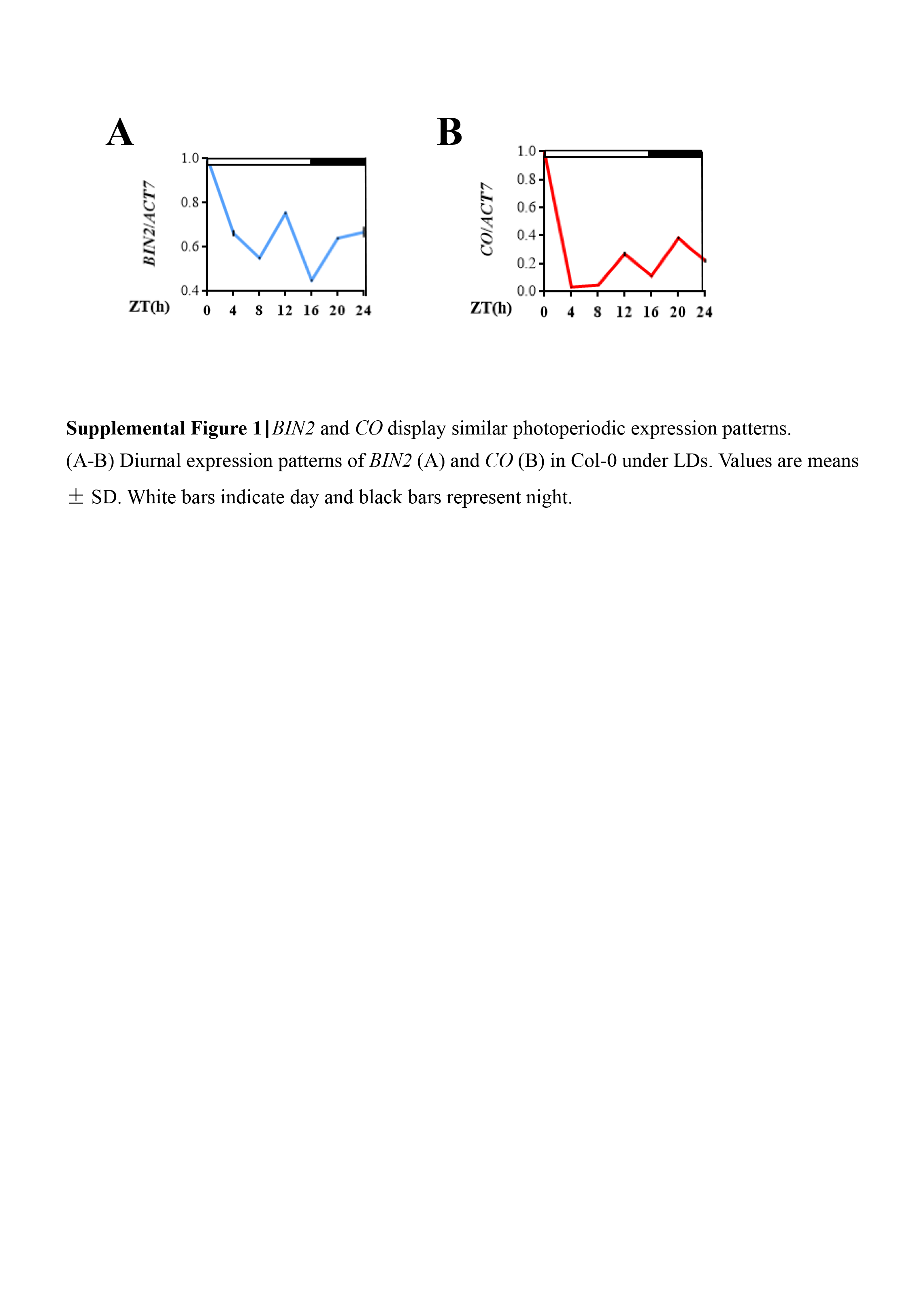

Supplement: Supplementary file 2 [file Image_1.tif]

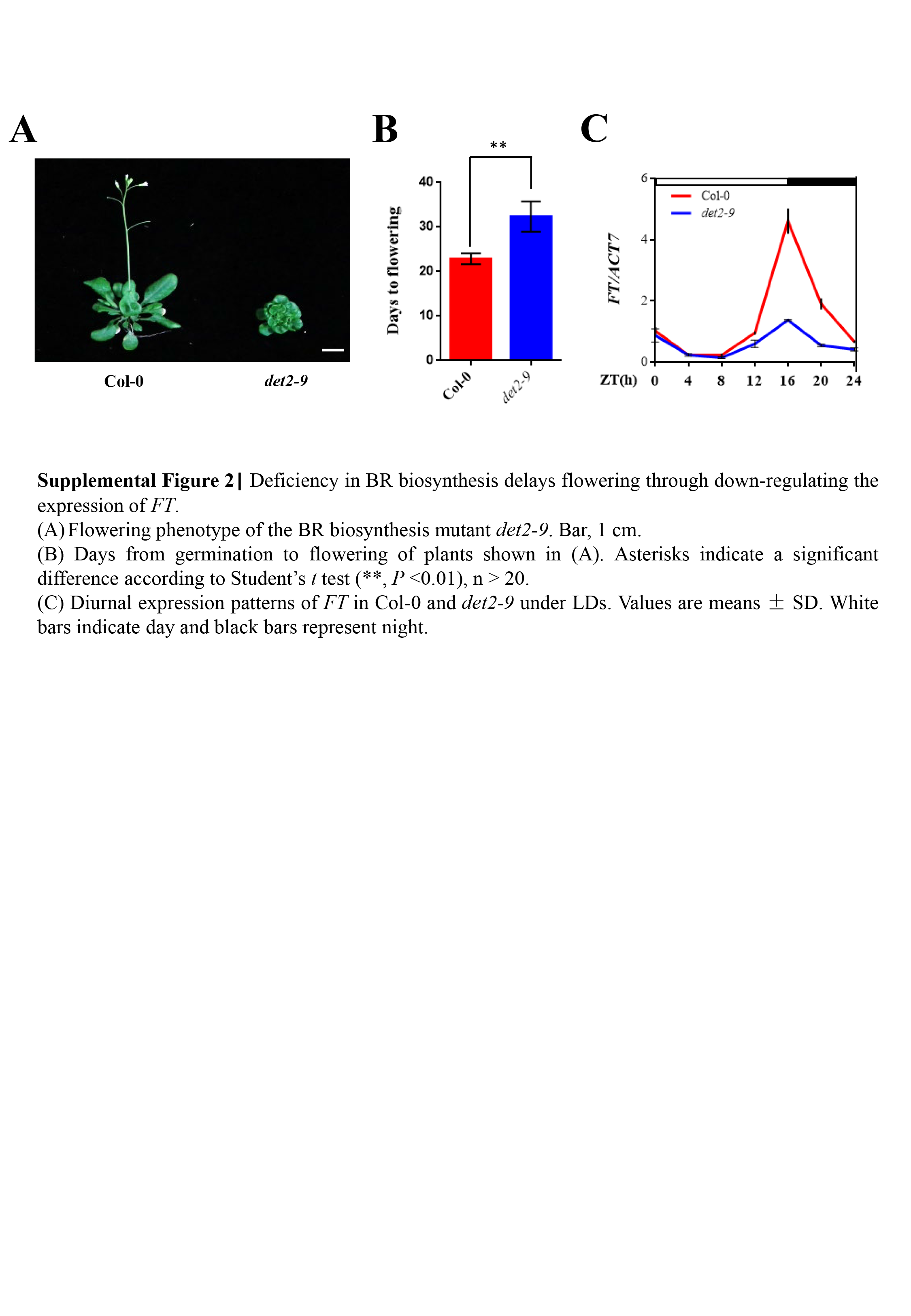

Supplement: Supplementary file 3 [file Image_2.tif]

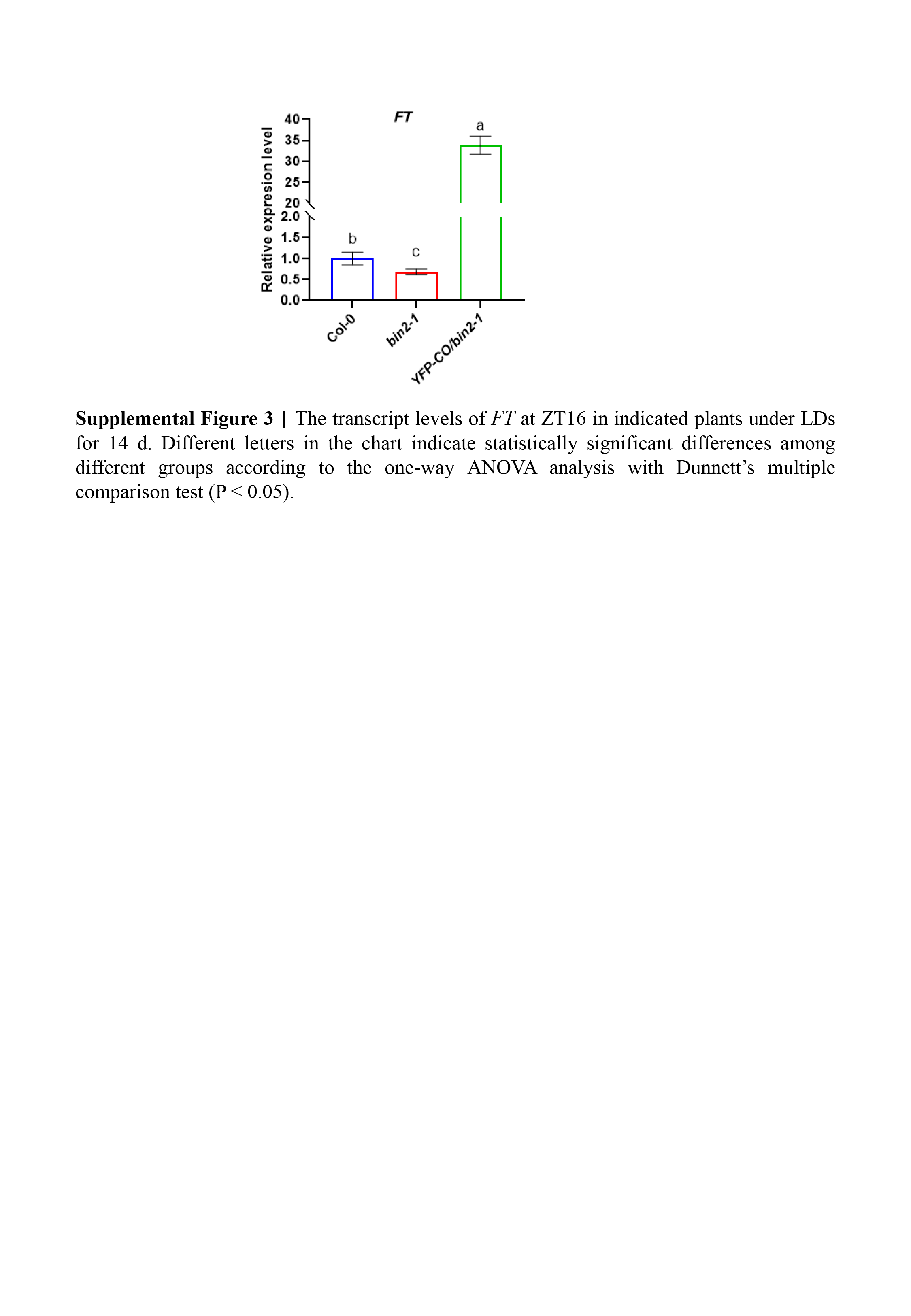

Supplement: Supplementary file 4 [file Image_3.tif]

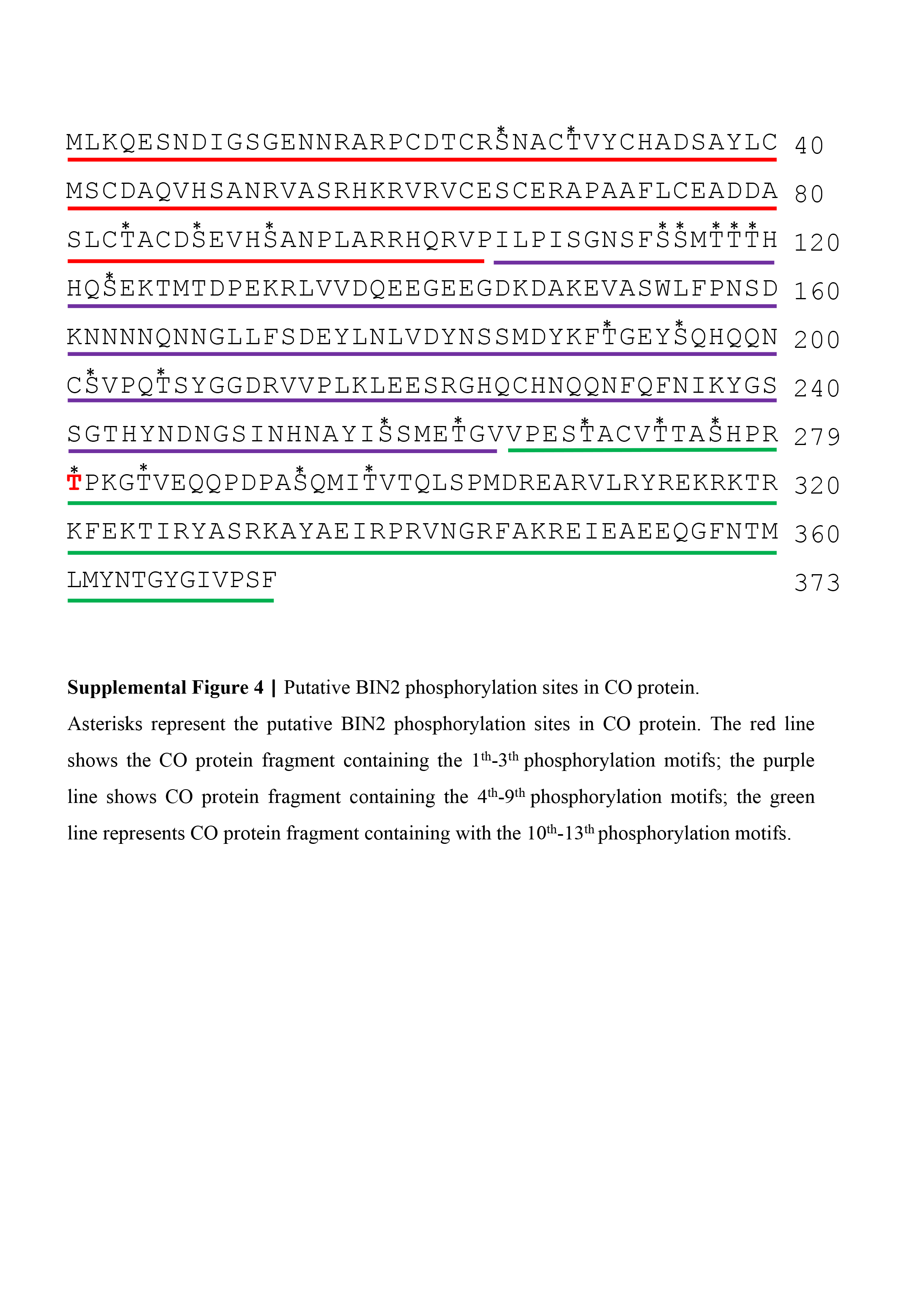

Supplement: Supplementary file 5 [file Image_4.tif]

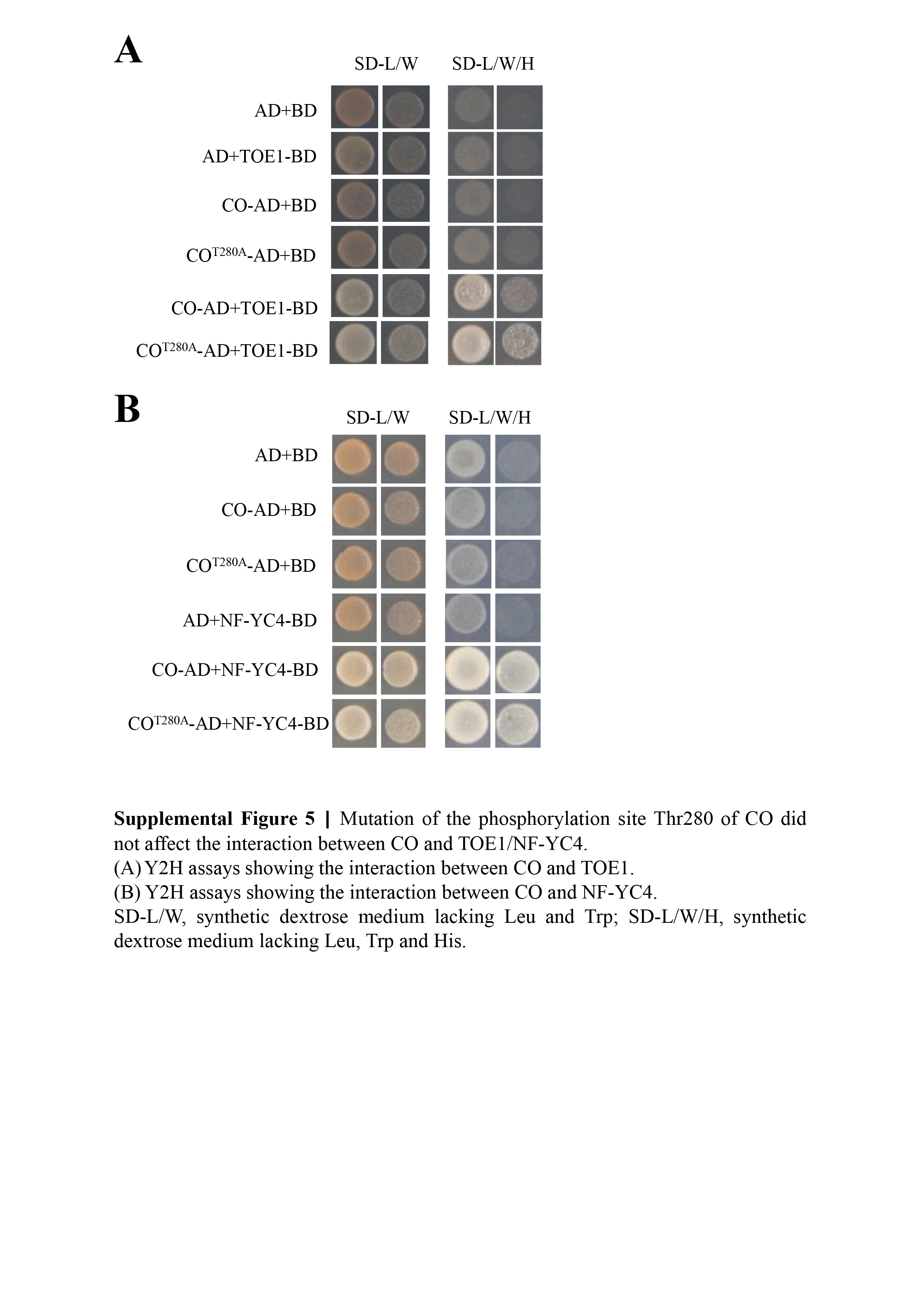

Supplement: Supplementary file 6 [file Image_5.tif]

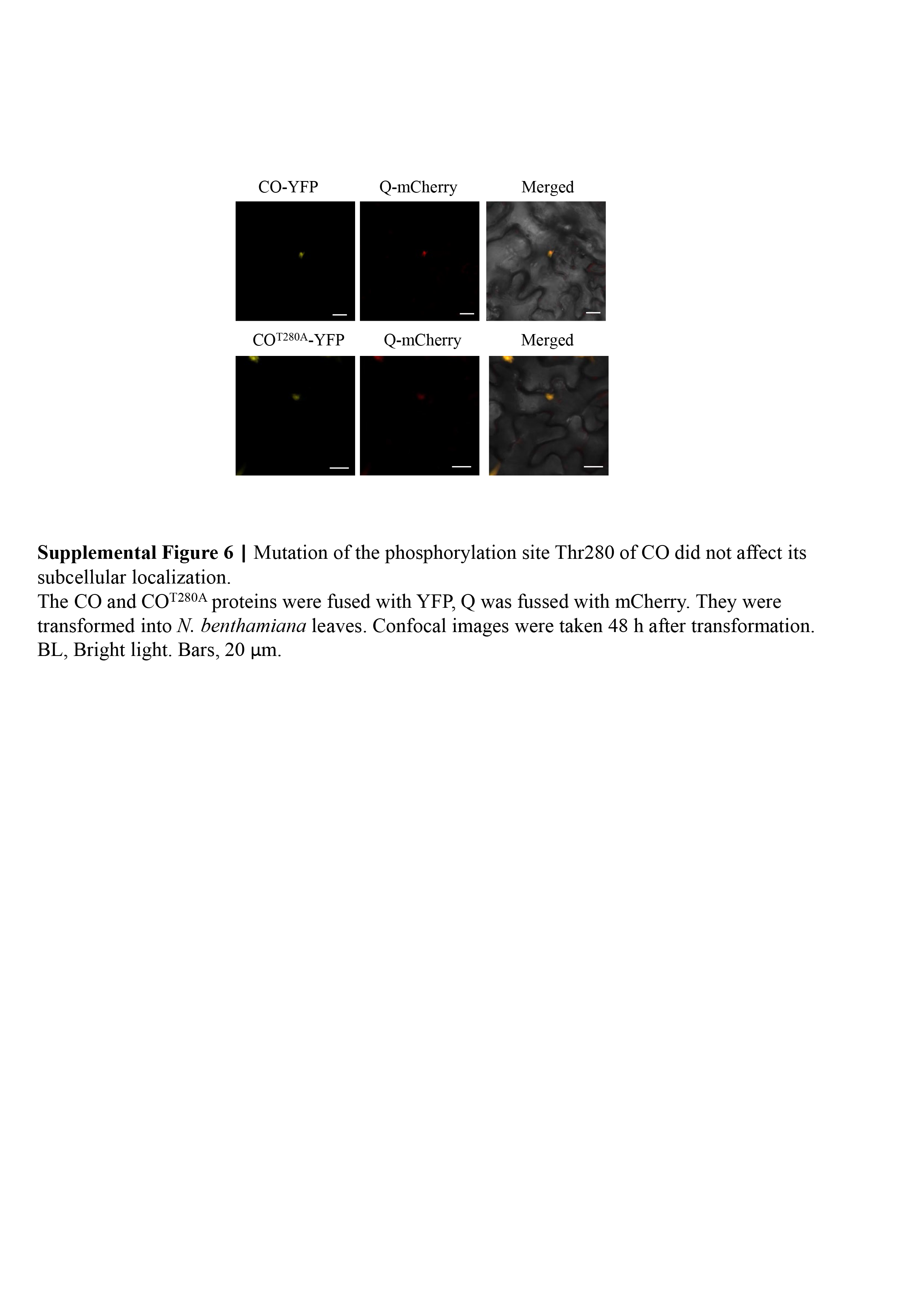

Supplement: Supplementary file 7 [file Image_6.tif]

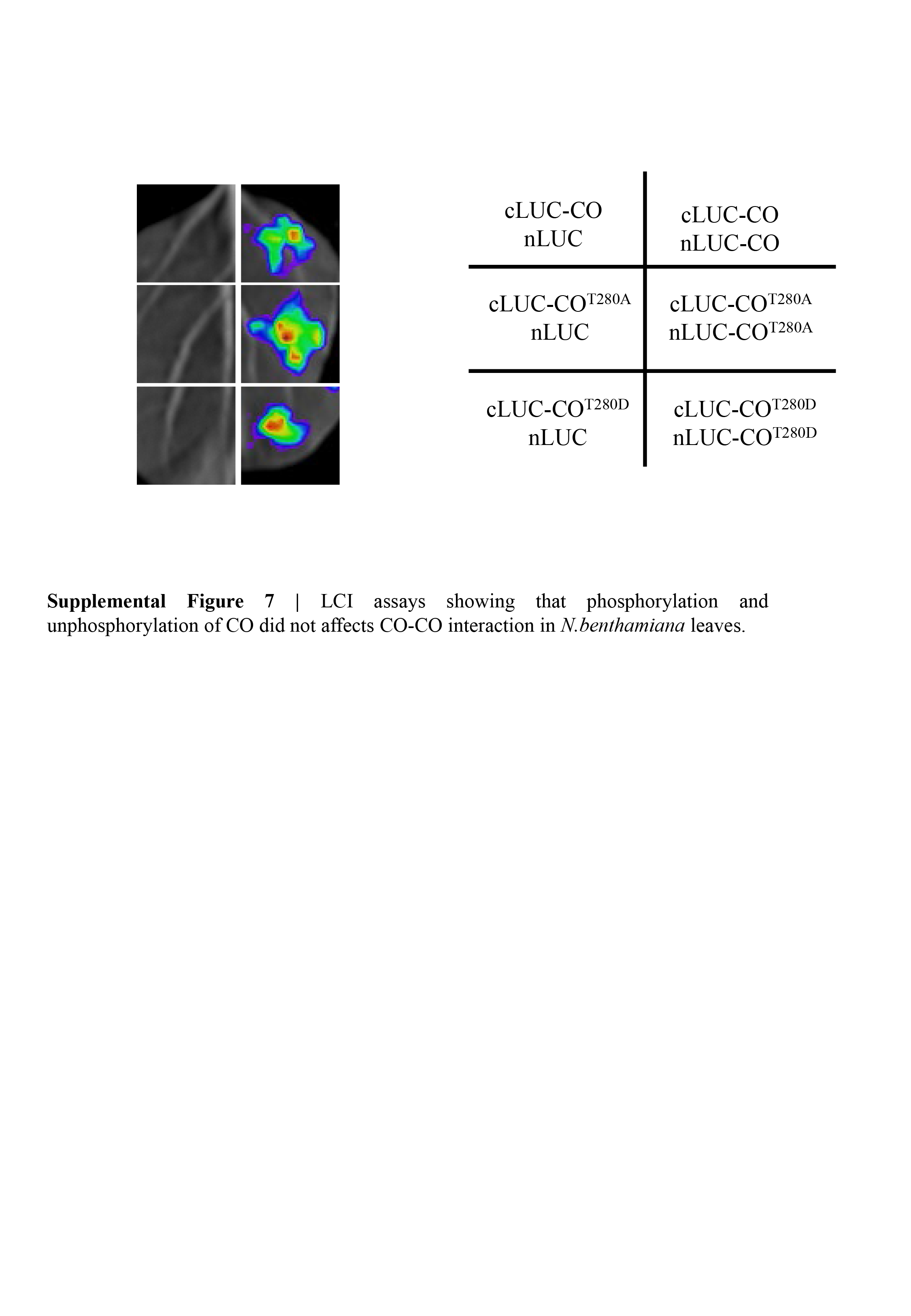

Supplement: Supplementary file 8 [file Image_7.tif]
